# Association of leisure sedentary behavior and physical activity with the risk of nonalcoholic fatty liver disease: a two-sample Mendelian randomization study

**DOI:** 10.3389/fnut.2023.1158810

**Published:** 2023-06-09

**Authors:** Xicheng Zhang, Keke Chen, Shangyu Yin, Mengao Qian, Changbao Liu

**Affiliations:** Department of Colorectal Surgery, The Second Affiliated Hospital and Yuying Children’s Hospital of Wenzhou Medical University, Wenzhou, China

**Keywords:** NAFLD, Mendelian randomization study, sedentary behavior, physical activity, causality

## Abstract

**Introduction:**

Previous observational studies have demonstrated the relationship between leisure sedentary behavior, physical activity, and nonalcoholic liver disease (NAFLD). However, whether these associations are causal or confounding factors remains unclear.

**Methods:**

Pooled genetic data from the UK Biobank and other large genome-wide association studies (GWAS) were used to extract instrumental variables representing sedentary television watching, computer use, driving, vigorous physical activity (VPA), and moderate-to-vigorous physical activity (MVPA). The two-sample Mendelian randomization (MR) method was used to explain the causal relationship between them and NAFLD. The inverse variance of the weighted method was used as the main analysis method, and MR-Egger, weighted median, MR-PRESSO, and other supplementary methods were also used. A sensitivity analysis was also performed. Simultaneously, the common risk factors for NAFLD were further analyzed for potential mediating associations.

**Results:**

We observed that sedentary television viewing (odds ratio (OR): 1.84; 95% confidence interval (CI): 1.09–3.10; *p* = 0.021) and genetically predicted VPA duration (OR: 0.0033; 95% CI: 0.000015–0.70; *p* = 0.036) were suggestively associated with the risk of NAFLD. Using a computer (OR: 1.51; 95% CI: 0.47–4.81; *p* = 0.484), driving (OR: 0.78; 95% CI: 0.05–11.94; *p* = 0.858), and MVPA time (OR: 0.168; 95% CI: 0.01–2.81; *p* = 0.214) were not significantly associated with NAFLD. The role of heterogeneity versus pleiotropy was limited in all the analyses.

**Discussion:**

This study supports the association between sedentary television watching and an increased risk of NAFLD, along with vigorous physical activity as a possible protective factor for NAFLD.

## 1. Introduction

Nonalcoholic fatty liver disease (NAFLD) is an important chronic liver disease worldwide. NAFLD is a general term for a series of diseases ranging from hepatic steatosis to nonalcoholic steatohepatitis and may even develop into cirrhosis and hepatocellular carcinoma, among which the most common type is NAFLD (1). The incidence of NAFLD has increased globally in recent years. In developed countries, it was the most common cause of chronic liver disease, and its prevalence and mortality rate due to liver disease are gradually increasing in many developing countries. The prevalence of NAFLD in China has even reached 29.2% (2).

Moreover, with the increasing prevalence of NAFLD, there is a corresponding increase in the economic and social burden on society. Research on risk factors can provide more information for the prevention of NAFLD. Previous observational studies have discovered that risk factors for NAFLD include obesity, metabolic abnormalities, smoking, drinking, and waist circumference (3, 4). However, the above factors still need to be further confirmed owing to the potential confounding and reverse causality inherent in observational studies.

Leisure sedentary behavior (LSB) refers to waking body behaviors that rely on lying down and sitting to maintain a low metabolic state (energy expenditure ≤ 1.5 metabolic equivalents) of body posture. This includes watching television (TV), using computers, driving, etc. Previous studies have discovered that prolonged sitting is associated with an increased risk of death from chronic diseases, whereas high physical activity (PA) levels can reduce this risk (5). In a study based on Physical Activity Guidelines for Americans, those who sitting more than 8 h/day were associated with increased risk for NAFLD, indicate that sedentary behavior is an independent predictor of NAFLD (6). A recent retrospective cohort study reported that regardless of confounding factors such as age, sex, energy intake, occupational PA, smoking, and alcohol consumption, sustained TV time was associated with a 2.3-fold increased risk of fatty liver disease (95% confidence interval (CI) 1.2–4.5) (7). Also, a study in China found that, the overall computer/mobile devices usage time levels and overall NAFLD, with a 1.99-fold (95% CI 1.29–3.05) increase in the prevalence of NAFLD in participants who viewed a screen ≥10 h/d compared to those who viewed a screen<1 h/d (8). PA cannot be ignored in terms of sedentary behavior, and the relationship between the two is very close. Long periods of sedentary behavior tend to indicate less PA. Many studies and reviews have shown that lack of PA and excess nutrition are the root causes of NAFLD (9). However, due to the lack of prospective studies on the relationship between LSB, PA, and the incidence of NAFLD, causal inference between LSB and NAFLD cannot be correctly established because retrospective studies are susceptible to potential confounding factors. Therefore, we inferred a causal relationship between LSB, PA, and NAFLD through a Mendelian-randomization (MR) study.

As an epidemiological method, MR has strong power to infer causality. It uses genetic variation as an instrumental variable (IV) for exposure and outcome, usually single-nucleotide polymorphisms (SNPs). The IVs are derived from large genome-wide association studies (GWAS) databases. However, these genetic variants are randomly assigned in deceleration division, which can reduce the influence of potential confounding factors and reverse causality and more directly prove the causal relationship between exposure and outcome (10, 11). Moreover, with the increase and accumulation of GWAS studies in recent years, the acquisition of MR-design data has become increasingly common and accurate.

In this study, we identified SNPs associated with NAFLD, LSB, and PA from a recent large GWAS dataset and explored the causal relationship between NAFLD, LSB, and PA using a two-sample MR method.

## 2. Materials and methods

### 2.1. Study design

**Figure 1:**
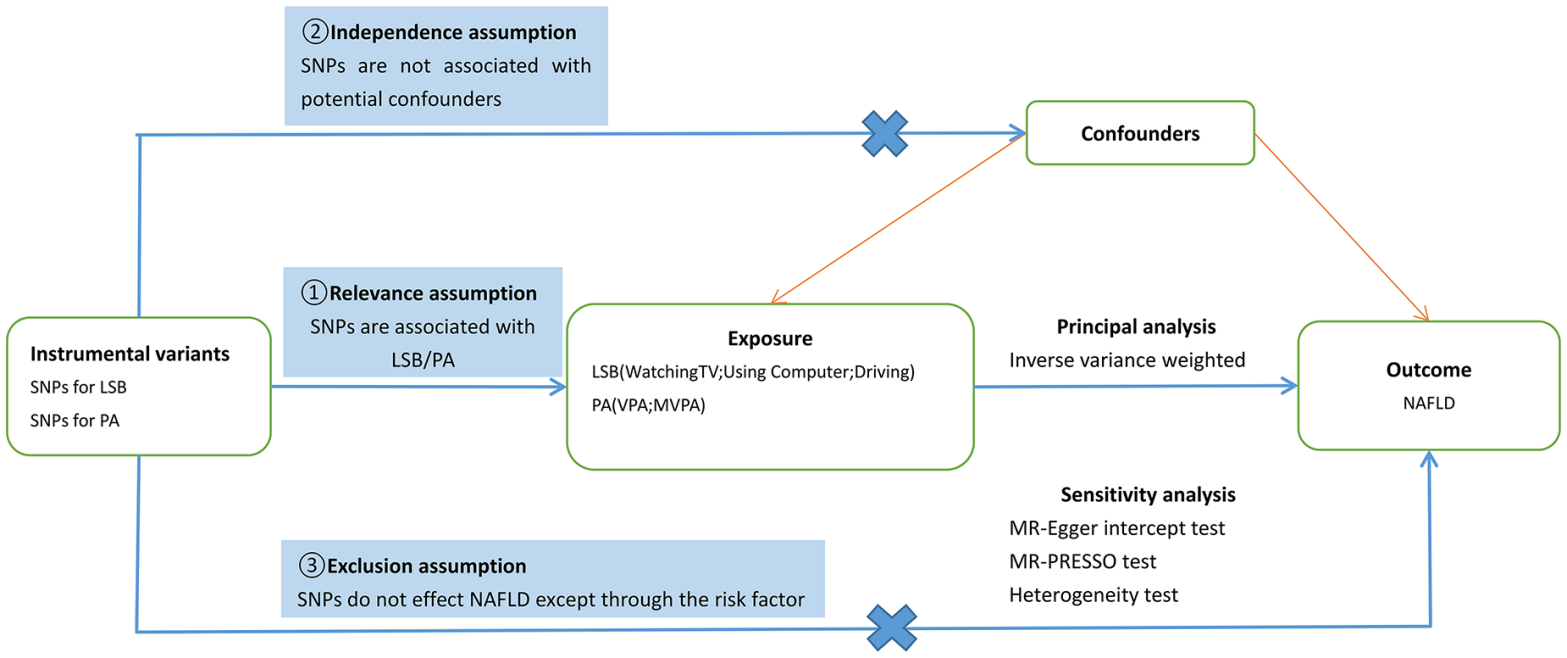
Schematic diagram of the present Mendelian randomization study. SNPs, single nucleotide polymorphisms; LSB, Leisure Sedentary Behavior; PA, Physical Activity; NAFLD, nonalcoholic fatty liver disease; VPA, vigorous physical activity; MVPA, moderate-to-vigorous physical activity; MR-PRESSO, MR Pleiotropy Residual Sum and Outlier.

SNPs were used as IVs for LSB in the MR study. Exposure and outcome SNP data were obtained from large GWAS databases. Three basic principles of MR research (Figure 1) were followed in the study design (12). The basic steps included the following: (1) selection of IVs related to exposure, (2) finding instrumental SNPs representing outcomes from GWAS datasets, (3) extracting and harmonising exposure and outcome data, (4) MR analysis, and (5) evaluation and analysis of MR results. All data in this study are publicly available, and relevant ethical approval and informed consent have been provided in previous studies.

### 2.2. Data sources of exposure samples

In this study, exposure included LSB and PA. The SNPs associated with sedentary behavior were identified from a recent large GWAS dataset of 422,218 European individuals; 45.7% were men, and 54.3% were women (13). LSB in this study included three specific behaviors: watching TV, using computers, and driving. Results were obtained in the form of a questionnaire in which participants answered the following questions: “How many hours do you spend watching TV on a typical day?,” “How many hours do you spend using the computer on a typical day?,” “How many hours do you spend driving on a typical day?.” The mean age of the participants was 57.4 years (standard deviation (SD): 8.0), and the data obtained showed an average of 2.8 h per day of TV watching (SD: 0.8), 1.0 h per day of recreational computer use (SD: 1.2), and 0.9 h per day of driving (SD: 1.0).

PA SNPs were derived from a UK Biobank GWAS study, which included 377,234 individuals from Europe (14). In this study, PA mainly included moderate-to-vigorous PA (MVPA) and vigorous PA (VPA). Relevant data were obtained using a touchscreen questionnaire, similar to the International Physical Activity Questionnaire (15). MVPA and VPA data were obtained by recording and calculating the responses to the following questions: “How many days did you do 10 min or more of vigorous physical activity in a typical week? (These are activities that make you sweat or breathe hard, such as fast cycling, aerobics, heavy lifting),” “In a typical week, how many days did you do 10 min or more of moderate physical activities like carrying light loads, cycling at a normal pace? (Do not include walking).”

### 2.3. IV selection

We screened for effective IVs using the following steps: (1) the genome-wide significance level was set at *p* < 5 × 10^−8^ to meet the first key hypothesis; that is, these SNPs were significantly associated with exposure (16), (2) Linkage disequilibrium clumping (*r*^2^ < 0.001, region size = 10,000 kb) was performed to ensure the independence of SNPs (17), (3) *R*^2^ and F-statistics were used to test the interpretation and strength of IVs, and the low-intensity IVs (F-statistics < 10) were removed. R^2^ = 2 × EAF × (1 − EAF) × betaˆ2/(2 × EAF × (1 − EAF) × betaˆ2) + 2 × EAF × (1 − EAF) × se × N × betaˆ2, F = R^2^ × (N−2)/(1−*R*^2^) (18, 19), (4) The phenotypes of SNPs were queried using PhenoScanner V2, and SNPs closely related to confounding factors (such as body mass index (BMI), body weight, waist circumference, hypertension, and other risk factors for NAFLD) were excluded to meet the second hypothesis (20). This guarantees the exclusivity of IVs. In this study, 36, 11, 0, 1, and 4 confounding SNPs associated with TV watching, computer use, driving, VPA, and MVPA, were removed, respectively. The specific information of the excluded SNPs is presented in Supplementary Table S2, and (5) For the third key hypothesis, we excluded SNPs that were correlated closely with the outcome (*p* < 5 × 10^−8^ (21)); Finally, 78, 22, 2, 5, and 4 SNPs were found to be associated with TV watching, computer use, driving, VPA, and MVPA. Detailed information regarding the SNPs is provided in Supplementary Table S3. For SNPs in the exposure data that could not be extracted from the outcome data, we used proxy SNPs with a cutoff *R*^2^ > 0.8. These SNPs were discarded if no proxies were identified.

### 2.4. Data sources of outcome samples

IVs for outcomes were derived from a large GWAS dataset of 1,483 European NAFLD cases and matching 17,781 controls. Their mean age was 50.1 (SD: 13.0); 47.3% were women, and 52.7% were men. The diagnostic criterion for NAFLD is pathological examination after liver biopsy (22).

In addition to exploring the relationship between LSB, PA, and NAFLD, the causal relationship between LSB, PA, and the risk factors for NAFLD was also studied. These risk factors mainly include triglyceride, LDL-C, HDL-C, type 2 diabetes, BMI, and hypertension. GWAS related to lipid traits, including triglyceride, LDL-C, and HDL-C, were obtained from the UK Biobank (23). The GWAS dataset associated with BMI and hypertension was obtained from the MRC Integrative Epidemiology Unit database. Related to diabetes, GWAS comes from DIAGRAM (24). All SNPs of the above NAFLD risk factors were searched for using the IEU OpenGWAS project platform.^1^ Ethical approval was obtained from each GWAS dataset. The specific information of the datasets is presented in Table 1.

**Table 1:** Detailed information of the studies included in the MR analyses.

Phenotype	Sample size	Consortium or author	Ethnicity	Year	Web source
LSB	422218participants	UK Biobank	European	2020	https://www.nature.com/articles/s41467-020-15,553-w
PA	377234participants	UK Biobank	European	2018	https://www.nature.com/articles/s41366-018-0120-3
NAFLD	1,483 cases and 17,781 controls	Anstee et al	European	2020	https://www.ebi.ac.uk/gwas/
LDL-C (mmol/L)	440,546participants	UK Biobank	European	2020	https://gwas.mrcieu.ac.uk/datasets/ieu-b-110/
HDL-C (mmol/L)	403,943participants	UK Biobank	European	2020	https://gwas.mrcieu.ac.uk/datasets/ieu-b-109/
Triglycerides (mmol/L)	441,016participants	UK Biobank	European	2020	https://gwas.mrcieu.ac.uk/datasets/ieu-b-111/
BMI	454,884participants	MRC-IEU	European	2018	https://gwas.mrcieu.ac.uk/datasets/ukb-b-2303/
Tpye 2 diabetes	69,033participants	DIAGRAM	European	2012	https://gwas.mrcieu.ac.uk/datasets/ieu-a-26/
High blood pressure	461,880participants	MRC-IEU	European	2018	https://gwas.mrcieu.ac.uk/datasets/ukb-b-14177/

NAFLD, nonalcoholic fatty liver disease; LSB, Leisure Sedentary Behavior; PA, physical activity; LDL-C, low density lipoprotein-cholesterol; HDL-C, high density lipoprotein-cholesterol; BMI, body mass index; MRC-IEU, MRC Integrative Epidemiology Unit.

### 2.5. Statistical analysis

In this MR Study, the primary statistical analysis method used was the inverse variance of the weighted method (IVW). It provides the main causal relationship between SNP exposure and outcome. Moreover, as long as SNPs are valid and do not show pleiotropy, IVW is the most commonly used and convincing MR statistical method (25). We also used the weighted median (26), MR-Egger (27), simple median, MR-RAPS (28), and MR-PRESSO (29) for statistical analysis. When there is heterogeneity among SNPs, IVW (multiplicative random effects) is more reliable than IVW (fixed effect) (30). The weighted median method estimates the MR for each IV by assuming that at least 50% of IVs are valid. The MR-Egger is mainly used to evaluate horizontal pleiotropy. When the intercept is off 0, there is directional pleiotropy, and when there is horizontal pleiotropy, the slope of the MR Egger regression is a relatively effective MR estimate (27). In the IVW analysis, MR-RAPS was corrected for horizontal pleiotropy using robust adjusted profile scores (28). We also used MR-PRESSO, which can identify outliers of pleiotropy, and then removed abnormal SNPs to obtain a relatively accurate MR estimate without bias (29). Finally, we used leave-one-out analysis to assess the effects of individual SNPs on the MR assessment.

To determine heterogeneity among IVs in this MR study, we mainly used Cochran’s Q and I^2^ statistics. The MR-Egger intercept, PhenoScanner V2, and MR-PRESSO global tests were used to determine pleiotropy between IVs. Power calculations were made using the online tool mRnd and based on the outcome data sample size, proportion of R2 sum, cases, and type I error rate of 0.05 (31). Taking into account multiple analyses, the Bonferroni-corrected value of *p* < 0.01 (0.05/5 exposures) was considered significant. *p*-values between 0.01 and 0.05 were considered suggested associations. All MR Analyses in this study were performed using R software (version 4.2.1) (32). The R packages used were TwoSampleMR (33) and MR-PRESSO.

## 3. Results

### 3.1. MR analysis of primary results

After the above screening steps, 11 SNPs could not be extracted from the NAFLD dataset among the 78 SNPs that represented watching TV. Among them, rs2184364, rs405797, rs55909997, rs66852340, rs7716447, rs7991062, and rs9569764 were replaced by rs4349826, rs178193, rs12401598, rs7668784, rs16867703, rs7335993, and rs956973 and other alternative SNPs. The remaining four SNPs were excluded from the analysis. Among the SNPs that represented computer use, rs11274218 and rs12874776 were replaced by rs12994113 and rs77640194, respectively, because they were not present in the NAFLD dataset. Rs166835 and rs2068625 were omitted because of a lack of relevant information. Rs35933007 was removed by MR-PRESSO because of its pleiotropic effect. Rs2854277, which represents MVPA, was excluded because no replacement could be found.

**Figure 2:**
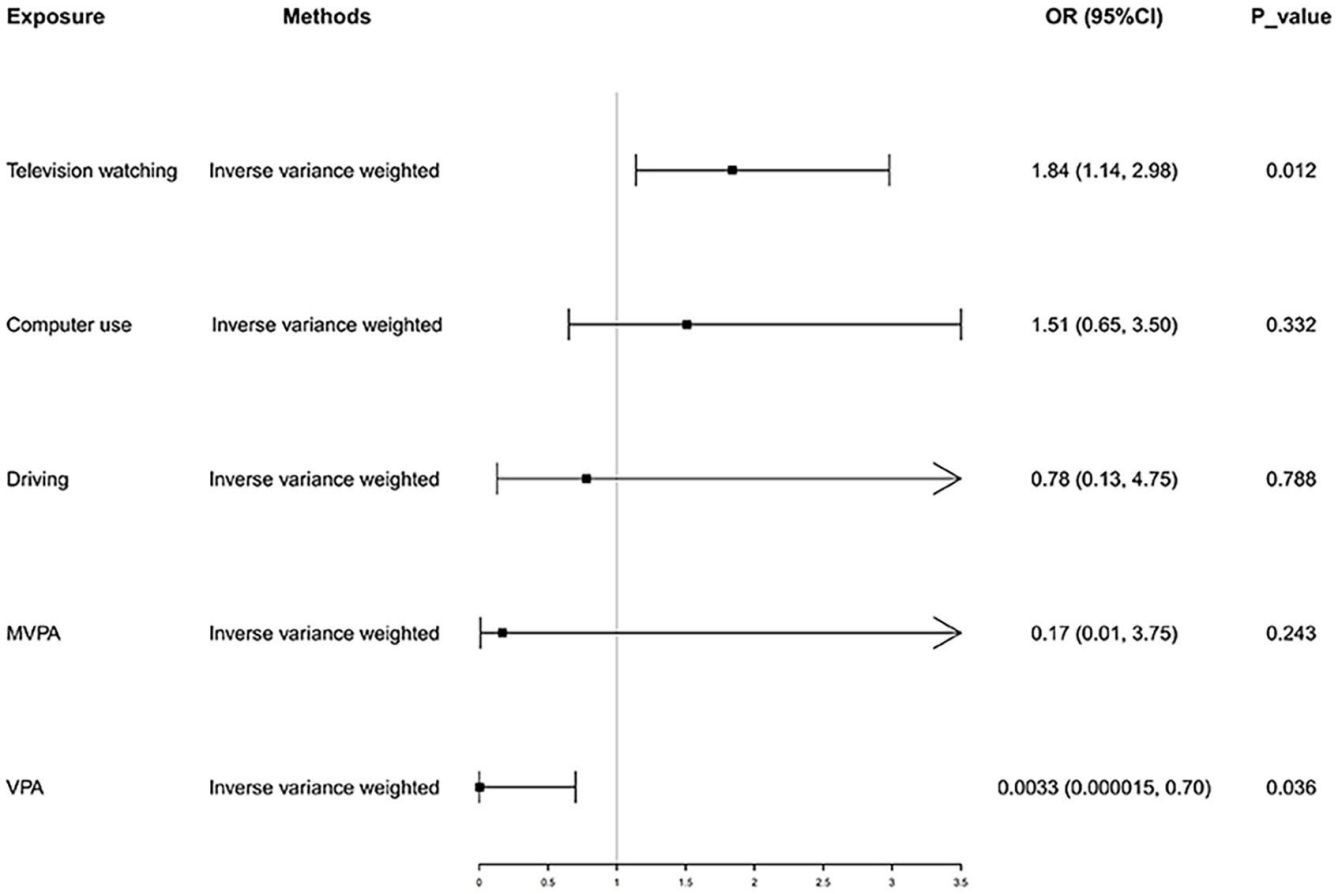
Causal effect of LSB and PA on NAFLD. NAFLD, nonalcoholic fatty liver disease; MVPA, moderate-tovigorous physical activity; VPA, vigorous physical activity.

**Figure 3:**
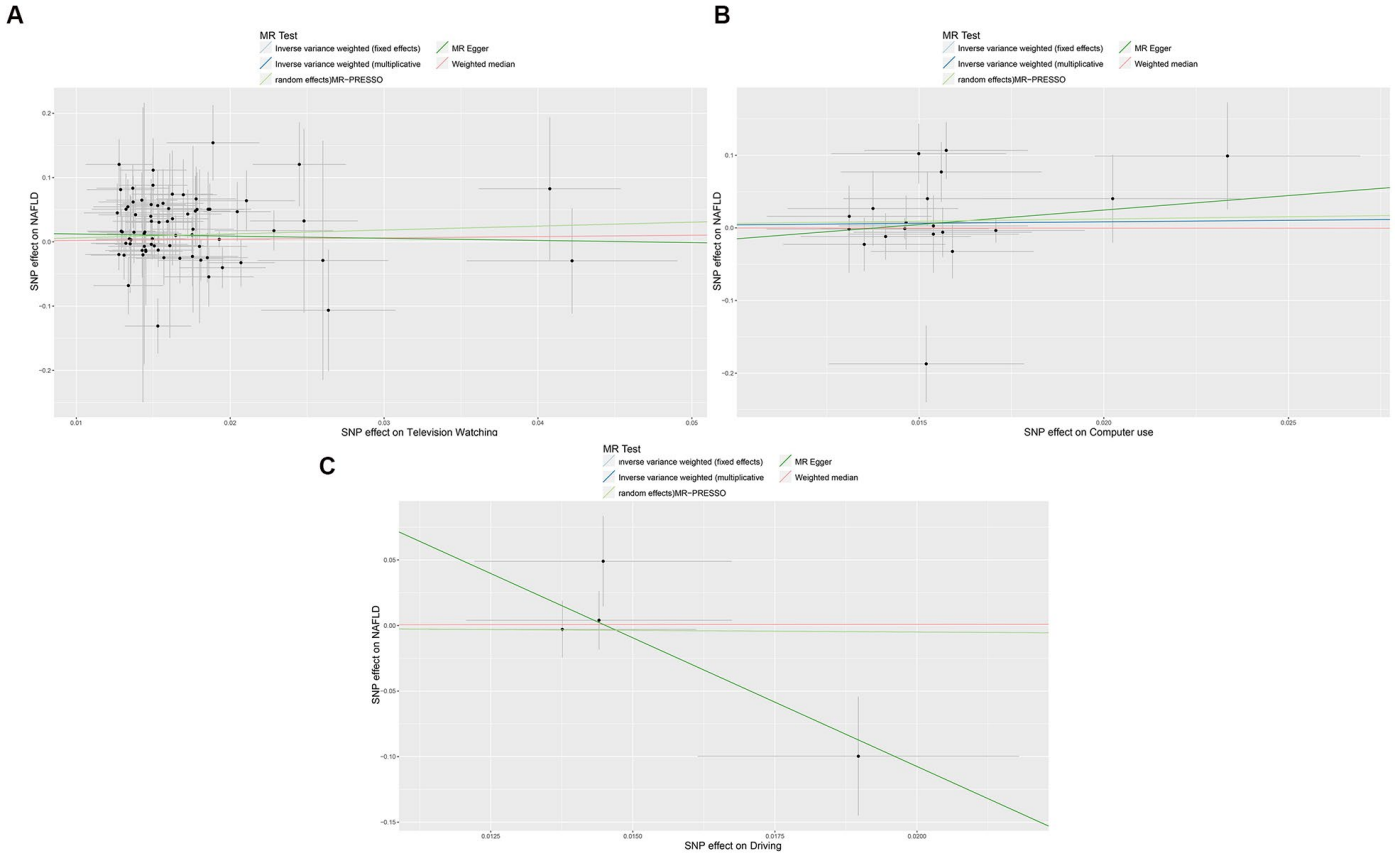
Scatter plots showing the genetic relationship between LSB with the risk of NAFLD, **(A)** television watching; **(B)** Computer use; **(C)** driving. NAFLD, nonalcoholic fatty liver disease; LSB, Leisure Sedentary Behavior. MVPA, moderate-to-vigorous physical activity; VPA, vigorous physical activity. MR-PRESSO, MR Pleiotropy Residual Sum and Outlier.

**Figure 4:**
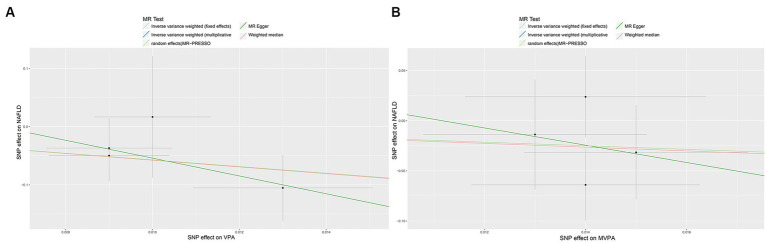
Scatter plots showing the genetic relationship between PA with the risk of NAFLD, **(A)** VPA; **(B)** MVPA. PA, physical activity; NAFLD, nonalcoholic fatty liver disease; VPA, vigorous physical activity; MVPA, moderate-to-vigorous physical activity.

In the IVW analysis, genetically predicted TV viewing time was suggestively associated with increased odds of NAFLD (odds ratio (OR): 1.84; 95% CI: 1.09–3.10; *p* = 0.021). Additional tests, such as MR-Egger, simple median, and MR-PRESSO, increased the stability of the results (Table 2 and Figure 2). However, the causal association between computer use and driving with NAFLD prevalence was not confirmed (computer use: OR: 1.51; 95% CI: 0.47–4.81; *p* = 0.484; driving: OR: 0.78; 95% CI: 0.05–11.94; *p* = 0.858). In the context of PA, genetically predicted VPA duration was suggestively associated with a reduced risk of NAFLD (OR: 0.0033; 95% CI: 0.000015–0.70; *p* = 0.036), indicating a lower risk of NAFLD in those who reported VPA activity on 3 or more days per week than in those who reported no VPA activity per week. However, it was not confirmed with MVPA (OR: 0.168; 95% CI: 0.01–2.81; *p* = 0.214) (Table 2 and Figures 3, 4).

**Table 2:** MR estimates of the causal association between leisure sedentary behaviors and PA with the risk of NAFLD.

Exposure	SNPs, *n*	Methods	NAFLD	
			OR (95% CI)	*p*
Television watching	74	IVW (random−effects)	1.84 (1.09,3.10)	0.021
	74	Weighted median	1.22 (0.58,2.59)	0.594
	74	MR − Egger	0.71 (0.04,12.62)	0.82
	74	MR − PRESSO*	1.84 (1.09,3.10)	0.024
Computer use	19	IVW (random−effects)	1.51 (0.47,4.81)	0.482
	19	Weighted median	0.98 (0.31,3.08)	0.978
	19	MR − Egger	53.59 (0.01,23727264.25)	0.556
	19	MR − PRESSO	1.84 (0.72,4.66)	0.214
Driving	4	IVW (random−effects)	0.78 (0.05,11.94)	0.858
	4	Weighted median	1.04 (0.10,10.43)	0.971
	4	MR − Egger	0.01 (0.01,10.04)	0.221
	4	MR − PRESSO*	0.78 (0.05,11.94)	0.869
MVPA	4	IVW (random−effects)	0.16 (0.01,2.81)	0.214
	4	Weighted median	0.15 (0.01,6.37)	0.324
	4	MR − Egger	0.01 (0.01,99)	0.853
	4	MR − PRESSO*	0.16 (0.01,2.81)	0.303
VPA	4	IVW (random−effects)	0.0033 (0.000015,0.70)	0.036
	4	Weighted median	0.0031 (0.0000015,1.64)	0.0712
	4	MR − Egger	0.001 (0.01,22808381.11)	0.45
	4	MR − PRESSO*	0.0033 (0.00018,0.06)	0.03

NAFLD, nonalcoholic fatty liver disease; SNPs, single−nucleotide polymorphisms; MVPA, moderate-to-vigorous physical activity; VPA, vigorous physical activity; OR, odds ratio; CI, confidence interval; IVW, inverse-variance weighted; MR-PRESSO, MR-pleiotropy residual sum and outlier; * means No outliers detected.

**Figure 5:**
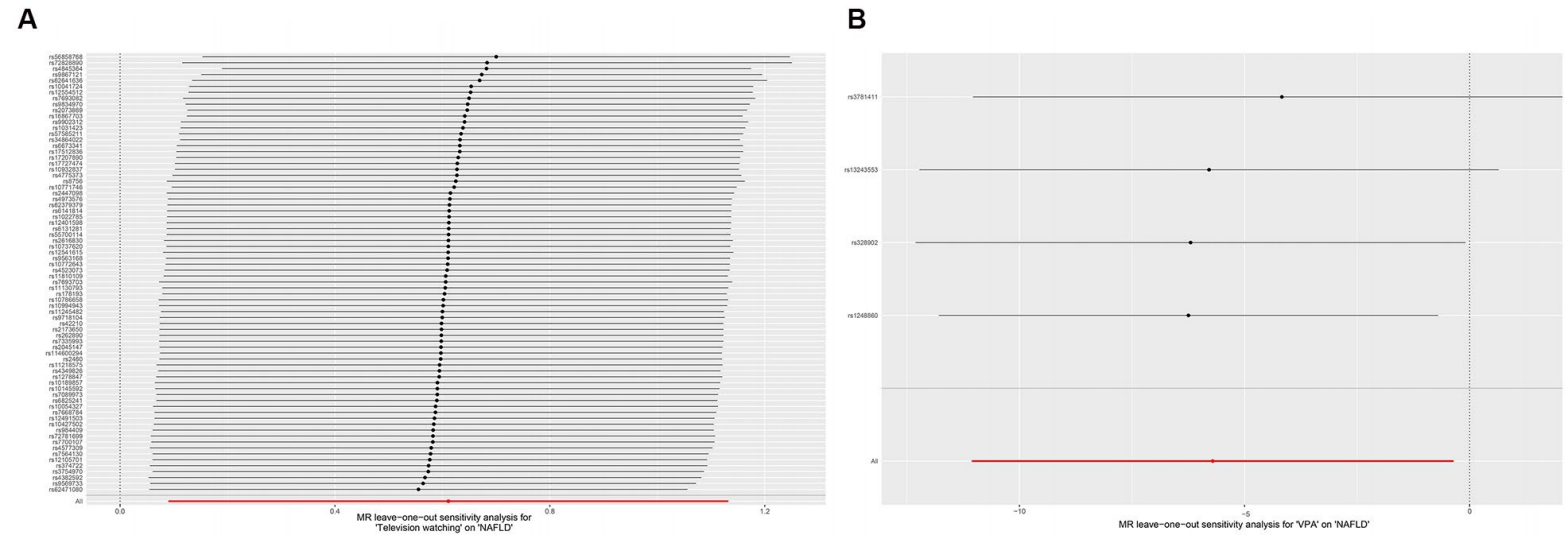
Plots of leave-one-out analyses for the causal effect of leisure television watching **(A)** and VPA **(B)** with the risk of NAFLD. VPA, vigorous physical activity; NAFLD, nonalcoholic fatty liver disease.

Heterogeneity was examined using Cochran’s Q and *I*^2^ tests. TV-watching-NAFLD (*p* = 0.137; *I*^2^ = 15.3%) and VPA-NAFLD (*p* = 0.830; *I*^2^ = 0) showed no significant heterogeneity among the IVs. Some heterogeneity was observed between computer use and driving in NAFLD (P_Cochran’s_ Q < 0.05, or *I*^2^ > 25%, Table 3) (34, 35). Pleiotropy was assessed by the MR-Egger intercept p and MR-PRESSO global test *p*. TV-watching-NAFLD (P_intercept_ = 0.513; P_global test_ = 0.153) and VPA-NAFLD (P_intercept_ = 0.613; P_global test_ = 0.85), and similar conclusions were identified in other results (Table 2) (29, 36). Leave-one-out analysis did not identify a single SNP that strongly influenced the causality between watching TV and NAFLD. However, there were two SNPs that strongly influenced the causal relationship between VPA and NAFLD (Figure 5). This study had 100 and 94% power, illustrating the causal relationship between watching TV and VPA and NAFLD, whereas the power between computer use, driving, MVPA, and NAFLD was 75, 8, and 68%, respectively.

**Table 3:** Heterogeneity and horizontal pleiotropy analyses of the primary MR study.

Outcome	Exposure	Heterogeneity test		Horizontal pleiotropy	
		*I*^2^(%)	Cochran’s Q p	MR-Egger Intercept *p*	MR-PRESSO Global Test *p*
NAFLD	Television watching	15	0.137	0.513	0.153
	Computer use	47	0.011^*^	0.596	0.684
	Driving	56	0.077	0.224	0.174
	MVPA	0	0.447	0.882	0.449
	VPA	0	0.83	0.613	0.85

NAFLD, nonalcoholic fatty liver disease; MVPA, moderate-to-vigorous physical activity; VPA, vigorous physical activity; MR − PRESSO, MR − pleiotropy residual sum and outlier; * means *p* < 0.05.

### 3.2. MR analysis of risk factors

We further explored whether there are potential mediating factors linking TV watching and VPA with NAFLD. We performed MR analyses of common risk factors for TV viewing and VPA with NAFLD. The results showed that one SD increase in TV watching time was associated with a 1.34-fold increase in BMI, a 1.07-fold increase in the risk of hypertension, a 19.4% decrease in HDL levels, 1.25-fold increase in triglyceride levels, and a 1.86-fold increase in the risk of type 2 diabetes. When vigorous PA increased by one SD, the risk of hypertension decreased by 19.1%, and there was no obvious causal relationship with BMI, TG, etc. (Table 4).

**Table 4:** MR analysis of leisure television watching and VPA with risk factors of NAFLD.

Outcome	Exposure	Number of SNPs	IVW (multiplicative random effects)		Heterogeneity		Pleiotropy	
			OR (95%CI)	*p*	Q value	*p*	Egger intercept	*p*
LDL-C	Television watching	62	1.03 (0.98, 1.09)	0.275	132.453	$3.33\times 10^{-7}$ ^*^	0.001	0.48
	VPA	4	1.06 (0.70, 1.59)	0.792	9.642	0.021^*^	−0.023	0.159
HDL-C	Television watching	62	0.81 (0.76, 0.86)	$1.54\times 10^{-11}$ ^*^	219.305	$1\times 10^{-19}$ ^*^	−0.003	0.263
	VPA	4	1.31 (0.93, 1.85)	0.12	8.109	0.043^*^	−0.006	0.714
Triglycerides	Television watching	62	1.26 (1.17, 1.35)	$5.99\times 10^{-10}$ ^*^	269.459	$5.37\times 10^{-28}$ ^*^	0.004	0.184
	VPA	4	0.70 (0.47, 1.06)	0.089	10.635	0.013^*^	0.003	0.885
BMI	Television watching	68	1.34 (1.24, 1.45)	$2.94\times 10^{-13}$ ^*^	383.548	$6.41\times 10^{-46}$ ^*^	0.003	0.333
	VPA	4	1.38 (0.72, 2.65)	0.331	26.785	$6.53\times 10^{-6}$ ^*^	−0.003	0.922
Type 2 diabetes	Television watching	32	1.86 (1.14, 3.05)	0.013^*^	39.994	0.129	−0.012	0.622
	VPA	4	2.11 (0.19,22.69)	0.539	4.686	0.196	0.101	0.339
Hypertension	Television watching	68	1.08 (1.04, 1.11)	$1.24\times 10^{-5}$ ^*^	312.614	$2.21\times 10^{-33}$ ^*^	0.001	0.414
	VPA	4	0.81 (0.72, 0.91)	0.0002^*^	3.793	0.285	−0.006	0.237

IVW, inverse variance weighted; OR, odds ratio; CI, confidence interval; LDL-C, low density lipoprotein-cholesterol; HDL-C, high density lipoprotein-cholesterol; BMI, body mass index; VPA, vigorous physical activity; * means *p* < 0.05.

## 4. Discussion

This two-sample MR Study provides strong and valid genetic evidence that leisurely sedentary TV watching leads to an increased risk of NAFLD and that increased VPA exposure reduces the risk for NAFLD. However, not all sedentary behaviors supported these results. Sedentary behaviors, such as computer use, driving, and MVPA, did not affect the risk of NAFLD. This finding was consistent with that of the primary MR analysis method (IVW) and other MR analyses and provided us with more genetic insights into the association between LSB and NAFLD.

Many studies have shown that sedentary behavior can increase the risk of obesity, hypertension, and other diseases (37, 38). Related animal studies have shown that PA alleviates the process of hepatic steatosis. Mechanistically, daily PA in rats prevents the development of hepatic steatosis and NAFLD by increasing the content and strengthening the function of liver mitochondria, and inhibiting hepatic lipogenesis (39). When the activity of rats was restricted, it was discovered that complete fatty acid oxidation in the liver and mitochondrial enzyme activities (citrate synthase, β-hydroxy-acyl-CoA dehydrogenase, and cytochrome oxidase) were decreased, while the liver contents of fatty acid synthase and acetyl-CoA carboxylase were increased. Although the phosphorylation status of acetyl-CoA carboxylase decreased, hepatic malonyl-CoA concentrations were significantly increased. However, these steps are all necessary for fatty acid synthesis and triglyceride production in the liver. Eventually, the incidence of fatty liver in rats increased (39). At the same time, a recent study on sedentary behavior and liver fat content also reported that liver fat content increased by 1.15% for every additional hour of sitting. These data can be understood by comparing the reduction in liver fat by 1.7% after 4 weeks of aerobic cycling intervention in obese men and women with sedentary habits (40). Our study also revealed similar results on the association between sedentary behavior, PA, and NAFLD. However, in this study, sedentary behavior limited to TV watching was associated with an increased risk of NAFLD, and the protective effect of PA on NAFLD was limited to VPA. This may be because watching TV is more relaxing than using computers and driving and requires less mental and limb activity. Studies have shown that leisure TV watching often means higher food and total energy intake compared with other sedentary behaviors such as reading, writing, and driving, which are usually induced by TV advertisements, and other unhealthy lifestyles, often replacing PA, thus increasing the risk of obesity (41, 42). Obesity is an identified risk factor for NAFLD. In this study, MVPA did not have the expected effect of reducing NAFLD risk, suggesting that walking, slow cycling, etc., may not have a significant protective effect against NAFLD. These findings strengthen the basis for further exploration and evaluation of the relationship between LSB, PA, and NAFLD.

In this study, we also analyzed the mediating reasons for the causal relationship between TV viewing and VPA and NAFLD. TV watching was associated with higher BMI, higher risk of hypertension, lower HDL levels, higher triglyceride levels, and a higher risk of type 2 diabetes. VPA reduces the risk of hypertension. Previous observational studies have shown that elevated BMI and blood lipid levels are clear risk factors for fatty liver disease (3). Previous studies have also demonstrated that the relationship between hypertension, diabetes, and NAFLD is often bidirectional (43). An Italian prospective study demonstrated for the first time that individuals with underlying hypertension had a nearly double risk of liver fibrosis progression (44). However, the association between diabetes and NAFLD is complex. Patients with type 2 diabetes show a high prevalence of NAFLD. In the Valpolicella Heart Diabetes study, the prevalence of NAFLD in nearly 3,000 patients with type 2 diabetes was 69.5% (45). Previous animal studies have also identified a relationship between the glucose-insulin pathway and NAFLD. Disruption of hepatic insulin signalling induces NAFLD and hepatic insulin resistance through insulin receptor substrate-2 gene deletion, leading to upregulation of SREBP-1, leading to the development of obesity, diabetes and NAFLD in laboratory animals (9). The above evidence demonstrates that BMI, blood lipid levels, hypertension, and diabetes may be mediating factors for NAFLD occurrence. TV watching may increase the risk of these risk factors and further affect NAFLD development. However, specific mediation analyses were lacking to determine the direct effects of TV watching and VPA on NAFLD. Nonetheless, this should not diminish the role of sedentary TV watching and VPA as etiological and protective factors for NAFLD. Reduced TV watching time and increased VPA time still affect NAFLD through possible intermediary factors.

This study used an MR analysis, which has the advantage of reducing residual confounding and reverse causality. Cochrane’s Q, MR-PRESSO global tests, and MR-Egger were used to examine the sensitivity of the results, and no heterogeneity or pleiotropy was observed among the main results. Simultaneously, the power of the two positive results exceeded 80%, which strengthened the stability of the results.

This study has some limitations. Firstly, it was difficult to stratify NAFLD for further study because the outcome data came from a large statistical GWAS. Secondly, some heterogeneity was identified in the study of mediating factors, which may be caused by different methods of data collection. Thirdly, although we performed sensitivity analyses that did not show substantial pleiotropy, we cannot rule out the possibility that horizontal pleiotropy may have biased the MR results. Fourthly, the power of the relationship between computer use, driving, MVPA, and NAFLD was 75, 8, and 68%, respectively, which did not reach the 80% threshold, possibly because the selected IVs had insufficient ability to explain computer use, driving, etc. Therefore, caution should be exercised when interpreting the association between computer use, driving, and NAFLD. Fifthly, there were two SNPs that strongly influenced the causal relationship between VPA and NAFLD in leave-one-out analysis. This may be due to too few SNPs in the study that are strongly associated with VPA, which requires larger GWAS studies to obtain more genetic data. Finally, since both exposure and outcome individuals were from Europe, it was difficult to interpret the results in the entire population. To our knowledge, this is the first MR study to explore the causal relationship between LSB and NAFLD using a large GWAS dataset. The study findings further prove that sedentary behavior contributes to an increased prevalence of NAFLD. At the same time, we also explored the mediating factors and observed that NAFLD is often the result of multiple factors. However, the specific proportion of mediating factors influencing NAFLD requires further study.

## 5. Conclusion

In conclusion, this MR Study demonstrated that more leisurely sedentary TV-watching time was associated with an increased risk of NAFLD, whereas more VPA time was associated with a corresponding reduction in NAFLD risk, although not at the level of significance after multiple testing correction. Although this study provides evidence for the prevention of NAFLD, the specific potential mechanism warrants more research.

## Data availability statement

The original contributions presented in the study are included in the article/Supplementary material, further inquiries can be directed to the corresponding author.

## Author contributions

XZ and KC: conceptualization, software, data curation, visualization, and manuscript writing. SY and MQ: writing review, visualization, and editing. CL: supervision of the study. All authors contributed to the article and approved the submitted version.

## Funding

This study was funded by the Wenzhou Municipal Science and Technology Bureau (Y20220888).

## Conflict of interest

The authors declare that the research was conducted in the absence of any commercial or financial relationships that could be construed as a potential conflict of interest.

## Publisher’s note

All claims expressed in this article are solely those of the authors and do not necessarily represent those of their affiliated organizations, or those of the publisher, the editors and the reviewers. Any product that may be evaluated in this article, or claim that may be made by its manufacturer, is not guaranteed or endorsed by the publisher.

## Footnotes

^1^https://gwas.mrcieu.ac.uk/

## Abbreviations

IVs: Instrumental variables
GWAS: genome-wide association study
VPA: vigorous physical activity
NAFLD: nonalcoholic fatty liver disease
TV: television
CI: confidence interval
OR: odds ratio
PA: physical activity
MR: Mendelian-randomisation
SNP: single-nucleotide polymorphism
LSB: leisure sedentary behavior
SD: standard deviation
MVPA: moderate-to-vigorous PA
VPA: vigorous PA
IVW: inverse-variance weighted.
